# Exploring the utility of *Brachypodium distachyon* as a model pathosystem for the wheat pathogen *Zymoseptoria tritici*

**DOI:** 10.1186/s13104-015-1097-9

**Published:** 2015-04-09

**Authors:** Aoife O’Driscoll, Fiona Doohan, Ewen Mullins

**Affiliations:** Department of Crop Science, Teagasc Research Centre, Oak Park, Carlow, Ireland; UCD Earth Institute and UCD School of Biology and Environmental Sciences, University College Dublin, Belfield, Dublin 4, Ireland

**Keywords:** *Brachypodium distachyon*, *Zymoseptoria tritici*, Septoria tritici blotch, wheat

## Abstract

**Background:**

*Zymoseptoria tritici*, the causative organism of Septoria tritici blotch disease is a prevalent biotic stressor of wheat production, exerting substantial economic constraints on farmers, requiring intensive chemical control to protect yields. A hemibiotrophic pathogen with a long asymptomless phase of up to 11 days post inoculation (dpi) before a rapid switch to necrotrophy; a deficit exists in our understanding of the events occurring within the host during the two phases of infection. *Brachypodium distachyon* has demonstrated its potential as a model species for the investigation of fungal disease resistance in cereal and grass species. The aim of this study was to assess the physical interaction between *Z. tritici* (strain IPO323) and *B. distachyon* and examine its potential as a model pathosystem for *Z. tritici.*

**Results:**

Septoria tritici blotch symptoms developed on the wheat cultivar Riband from 12 dpi with pycnidial formation abundant by 20 dpi. Symptoms on *B. distachyon* ecotype Bd21-1 were visible from 1 dpi: characteristic pale, water soaked lesions which developed into blotch-like lesions by 4 dpi. These lesions then became necrotic with chlorotic regions expanding up to 7 dpi. Sporulation on *B. distachyon* tissues was not observed and no evidence of fungal penetration could be obtained, indicating that *Z. tritici* was unable to complete its life cycle within *B. distachyon* ecotypes. However, observation of host responses to the *Z. tritici* strain IPO323 in five *B. distachyon* ecotypes revealed a variation in resistance responses, ranging from immunity to a chlorotic/necrotic phenotype.

**Conclusions:**

The observed interactions suggest that *B. distachyon* is an incompatible host for *Z. tritici* infection, with STB symptom development on *B. distachyon* comparable to that observed during the early infection stages on the natural host, wheat. However first visible symptoms occurred more rapidly on *B. distachyon*; from 1 dpi in comparison to 12 dpi in wheat. Consequently, we propose that the interaction between *B. distachyon* and *Z. tritici* as observed in this study could serve as a suitable model pathosystem with which to investigate mechanisms underpinning an incompatible host response to *Z. tritici*.

## Background

Wheat is grown on more land area than any other commercial crop in the world and it is the third most produced global cereal after rice and maize. Wheat is susceptible to multiple pathogens, including *Z. tritici,* the causal agent of Septoria tritici blotch (STB) disease. This disease requires intensive chemical control to protect yields. The cost to U.S. growers is estimated at ~ $275 million annually (http://jgi.doe.gov/why-sequence-mycosphaerella). In Europe, STB represents the greatest threat to wheat production with approximately 70% (> €400 M) of annual fungicide input directed towards preventing or reducing STB induced losses [1].

The physical infection process of wheat by *Z. tritici* has been previously well characterised [2-6]. With no specialised structures such as appressoria or haustoria for penetration of host tissues [4], *Z. tritici* infects solely via host stomatal cavities, with penetration occurring within 12 h (h) post-inoculation. Infecting hyphae grow exclusively intercellularly, advancing into the mesophyll layer with a minimal increase of *in planta* fungal biomass required before the onset of host cell death [7,8]. Between 12–20 dpi, *Z. tritici* switches to an aggressive necrotrophic lifestyle that leads to the characteristic lesions bearing spore-producing pycnidia along the leaf surface. Wheat promptly recognises the presence of *Z. tritici*, with transcriptional changes in defence-gene expression (e.g., PR proteins) recorded from as early as 6 h after inoculation [9], as well as rapid changes in host cell reactive oxygen species (ROS) during interactions with both virulent and avirulent isolates of the pathogen [6-8]. Mechanisms of susceptibility to *Z. tritici* are now better understood, with susceptible interactions acting as a primary source of information to date [6-13]. Evidence from such studies suggest that *Z. tritici* requires the activation of host cell death signalling pathways and disease lesion formation to acquire sufficient energy to reproduce asexually [6,7,11]. Yet despite extensive studies, hypersensitive response (HR)-like cell death has not been associated with a resistant interaction [2,4,6,9] as confirmed by the classic symptoms of PCD occurring during symptom development in the susceptible interaction but not so in the resistant [7,11]. It has been hypothesised that the lack of such activity in the resistant interaction is a consequence of more powerful upstream defence responses [1]. If so, what are these responses and do they occur during the early, biotrophic phase of this host-pathogen interaction?

Fully elucidating the host responses of wheat to *Z. tritici* is crucial to the understanding of how resistance, or lack thereof, to the pathogen operates. However because of its complex genome and long generation time, it remains a challenge to conduct in depth ‘omic’ studies in wheat [14]. Although, rapid developments have been made in the sequencing of the wheat genome [15,16], a deficit in genome annotation means that the interaction between wheat and *Z. tritici* will remain difficult to characterise genetically for the immediate future. A model pathosystem in which the host has been genetically characterised and is amenable to *Z. tritici* infection could act as an integral resource in the quest to answer some of the essential questions surrounding the manifestation of host resistance to *Z. tritici*. The insight gained using *Arabidopsis thaliana* as a model plant has allowed fundamental questions to be answered for a range of host-pathogen interactions [17-19] but to date no evidence exists that this plant can act as a host for *Z. tritici.*

*B. distachyon* has the potential to serve as such an alternative model system, having diverged from the Triticae between 35 and 40 million years ago (MYA) [20]. Although the closest relative to wheat is barley, which diverged from the Tritici between 10–15 MYA [21], *B. distachyon* remains more closely related to the Triticae family than rice or other sequenced grasses such as sorghum and maize, which diverged from the Triticae ~ 50 MYA [22]. *B. distachyon* has been described as a model plant for functional genomics in grasses for several reasons [23]: it possesses a relatively small genome with very little repetitive DNA, exhibits a low degree of DNA methylation and has a nuclear genome indistinguishable in size to that of *A. thaliana*, making it the simplest described genome of the grasses. Other desirable physiological attributes include a small stature, a short lifecycle, simple growth and handling requirements and an established transformation system [24]. In addition, an international effort has ensured that adequate resources for *B. distachyon* have been developed to provide seed stocks and genomics resources (www.brachypodium.org). Owing to these developments, the number of publications detailing the interaction between *B. distachyon* and many of the world’s most economically important cereal pathogens, including *Fusarium graminearum*, *Puccinia striiformis*, *Magnaporthe grisea*, *Bipolaris sorokiniana,* Oculimacula spp., *Ramularia collo-cygni* and most recently *Staganospora nodorum* has risen steadily over the last ten years [14,24-32]. The aim of this study was to assess the prospect of employing *B. distachyon* as a tractable pathosystem with which to comparatively investigate host defence mechanisms in the wheat-*Z. tritici* interaction.

## Methods

### Fungal material

The *Z. tritici* (teleomorph *Mycosphaerella graminicola*) isolate IPO323 has proven to be the isolate of choice for many host-pathogen studies conducted to date [4,6,8,10,11,13,33,34] and its preference as a reference isolate is evident by the publication of its sequenced genome in 2011 [35]. For this study, pycnidiospores of isolate IPO323 were stored at −70°C in 10% glycerol (v/v^−1^). Prior to use, the isolate was cultured on potato dextrose agar (PDA) and grown at 18°C under a 12 h light/ 12 h of near ultraviolet (NUV) darkness at 18°C for 7 days. Fungal spores for plant inoculation were harvested from these cultures into a 0.05% Tween 20 solution (v/v ^-1^ sterile distilled water (SDW)) before the spore concentration was adjusted to 1 x 10^6^ ml^−1^.

### Plant material and ecotypes

Seed of five *B. distachyon* inbred lines, designated by the prefix ‘’Bd” were provided by Dr. Carl Ng (University College Dublin) and Prof. David Garvin (University of Minnesota). These inbred lines; Bd1-1, Bd12-3, Bd21-1, Bd29-1 and Bd30-1 were developed by single seed descent from NPGS accessions obtained from the USDA Western Regional Research Centre in Albany, CA and the USDA Midwest Area, Plant Science Research Unit at the University of Minnesota, St. Paul, MN.

### Disease trials

For cultivation, seeds were stratified at 4°C for 5 days (with the exception of Bd1-1 where seeds were stratified for 8 weeks) then incubated in darkness at 23°C for 5 days to allow germination. Germinated seeds were transferred to 8 x 8 x 8 cm pots and placed in a controlled environment glasshouse, under a 16 h day/8 h night cycle at 23/12°C, respectively. One week prior to inoculation with the *Z. tritici* isolate IPO323; plants were transferred to an 18/12°C environment to allow acclimatisation to the optimum temperature required for *Z. tritici* infection. Plants were watered every 2 days and each day of the week before planned inoculations took place.

Once plants had reached the five leaf stage, whole *B. distachyon* plants were sprayed with a pycnidial suspension of IPO323 amended with a 0.05% Tween 20 solution (~5 ml) using a hand-held spray bottle. Control plants were inoculated with the same volume of a 0.05% Tween 20 solution. Treated plants were covered with polythene bags for 7 days to ensure high humidity. For initial studies on Bd21-1, experiments were conducted three times with 7 technical replicates (a technical replicate represented the fifth leaf of an individual plant) in each experiment. For studies on variations within ecotypes, the experiment was conducted three times with 8 technical replicates for each ecotype. Symptom progression was recorded at 1, 4 and 7 dpi with reaction types classified as (i) no symptoms, (ii) patches of yellow and red chlorosis, (iii) chlorosis with necrotic lesions or spots or (iv) necrosis with no symptoms of chlorosis, since the physiology of observed symptoms varied according to the ecotypes screened. The wheat variety Riband, inoculated at the two leaf stage was included as a positive control in initial experiments with Bd21-1 to ensure *Z. tritici* spore viability and isolate virulence. The sole alteration to experimental conditions for Riband was that the bags were left on for 14 days to maintain humidity to support infection. Disease symptoms were recorded every 2–3 days from 12 dpi up to 31 dpi.

In attempts to re-isolate *Z. tritici* from infected leaf tissues, two different procedures were used. Eight inoculated leaves from each ecotype were detached from whole individual plants at 14 dpi by which time they had completely senesced and no further signs of symptom progression could be observed. The leaves were surface sterilised in 70% ethanol for 10 sec followed by 5% sodium hypochlorite for 1 minute and rinsed three times in SDW. Leaves were gently dried using a paper towel and placed on water agar amended with streptomycin (50mgl^−1^) and penicillin (50mgl^−1^) to eliminate bacteria and mold contamination according to [36]. Leaves were incubated under 12 hours of NUV at 18°C for 96 hours, to encourage cirri emergence. Using a Zeiss Stemi DV4 light microscope and a sterile needle, leaves were examined for globose structures resembling pycnidia. In the second pursuit, the experimental design, sterilisation and incubation of the leaves were the same, however instead of attempting to isolate pycnidia using a microscope, sterile leaves were centrifuged at13,000 g in a 2 ml Eppendorf tube containing 50ul of SDW, for 30 seconds. The resulting pellet was streaked onto the surface of a PDA plate amended with the above antibiotics. The plates were sealed with parafilm and incubated for seven days after which they were examined for any evidence of Z*. tritici* growth.

### Microscopic analysis

Bd leaf sections were cleared in 70% ethanol at room temperature for 24 h to remove chlorophyll. To detect H_2_O_2_, leaf samples were placed in 1 mg ml^−1^ 3, 3’ diaminobenzadine (DAB-HCL) (Sigma Aldrich, UK) pH 3.8, according to the protocol described by [6]. For observations of fungal structures, cleared leaf samples were stained at room temperature for four hours in Trypan blue (Sigma Aldrich, UK) in lactoglycerol (1:1:1, lactic acid: glycerol: H_2_O_2_) and rinsed in chloral hydrate (2.5 g ml^−1^). Samples were mounted in 40% glycerol and examined for the presence of hyphal penetration within stomata. Thirty individual leaf samples were examined with 10 stomata assessed per inoculated leaf and viewed across three separate focal planes to detect for evidence of fungal attachment and/or entry into the stomatal cavity. Samples were viewed with a Leica DM 2500 microscope and photographed with a Sony AS400 digital camera.

## Results

**Figure 1:**
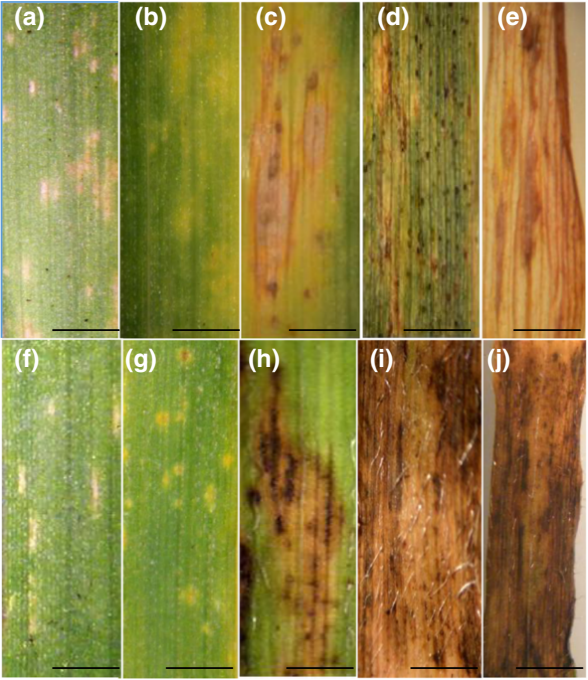
**Septoria tritici blotch symptoms on the wheat cultivar Riband and the** ***B. distachyon*** **ecotype Bd21-1. (a-e)** Symptoms were recorded on wheat at 12, 14, 17, 20 and 31dpi respectively. **(f-j)** Symptoms were recorded on Bd21-1 at 1, 4, 7, 14 and 21 dpi. Scale bars = 0.5 cm.

**Figure 2:**
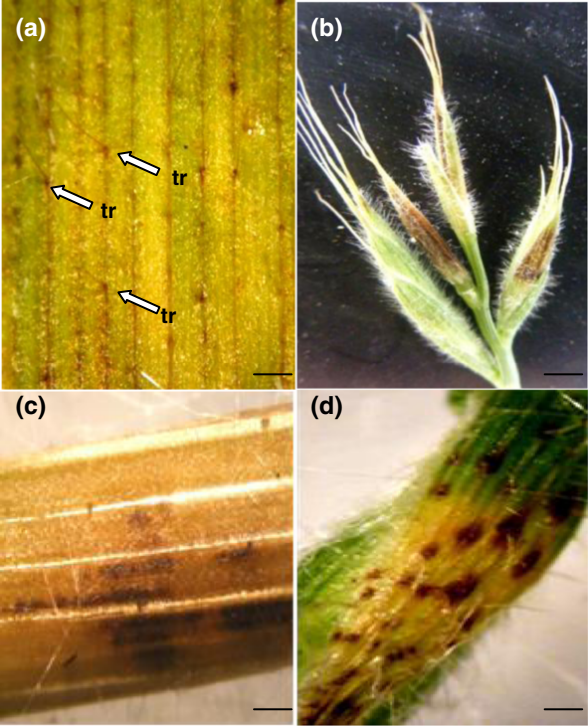
**Septoria tritici blotch symptoms on aerial tissues of the** ***B. distachyon*** **ecotype Bd21-1. (a-d)** Infection symptoms were visible as chlorotic/necrotic phenotypes on trichomes (tr), floral heads, stems and stem nodes respectively. Scale bars = 0.5 cm.

The wheat cultivar Riband developed pale water soaked lesions at 12 dpi, becoming chlorotic by 14 dpi (Figure 1a, b). Necrotic lesions bearing pycnidia were visible from 17 dpi (Figure 1c), with pycnidial formation abundant by 20 dpi (Figure 1d), continuing to develop up to 31 dpi by which time the leaf had fully senesced (Figure 1e). Leaves of Bd21-1 demonstrated pale, water soaked lesions at 1 dpi (Figure 1f). By 4 dpi, these lesions had become chlorotic in appearance with brown necrotic blotch like regions appearing at 7 dpi (Figure 1g, h). Lesions continued to expand across the leaf surface up to 14 dpi (Figure 1i). By 21 dpi, leaves had fully senesced, bearing tissue which was almost completely necrotic (Figure 1j). The symptoms on *B. distachyon* were eminently comparable to those observed during the early biotrophic phase of *Z. tritici* infection (Figure 1, compare panels a-e to panels f-j). STB symptoms were observed on all aerial parts of the *B. distachyon* plant including the trichomes, florets, stems and stem nodes (Figure 2a-d respectively). No evidence of other disease symptoms from other foliar pathogens were observed in the wheat or Brachypodium controls (data not shown).

**Figure 3:**
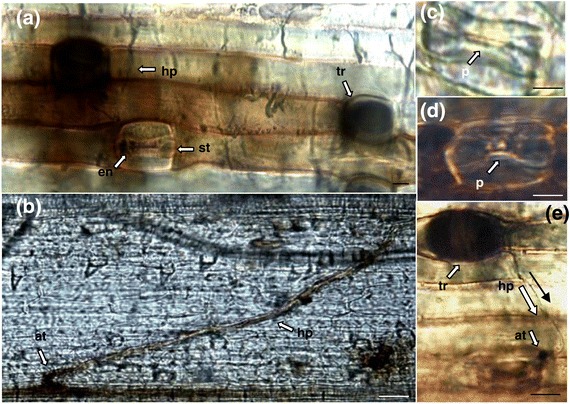
**Light microscopy analysis of leaves of the** ***B. distachyon*** **ecotype Bd21-1, cleared and stained with Trypan Blue and DAB. (a)** Fungal hyphae visible on leaf surface (hp), with H_2_O_2_ released from cells surrounding a stomata (st), Trypan blue staining is localised in the pore of the stomata (en, st) indicating the presence of fungal tissue. The bases of trichomes (tr) also stain heavily for necrotic tissue. **(b)** Hyphal attachment (at) at the junction between adjacent vascular tissues showing H_2_O_2_ production within the hyphae (hp) and at the site of attachment. Scale bars (a and b) = 0.5 mm **(c)** Uninfected stomata with clear pore (p) and no evidence of fungal staining. **(d)** Stomata with clear pore (p) and has not stained for fungal tissue, however H_2_O_2_ is present around the circumference of the stomata. **(e)** Fungal hyphae (hp) originating from the base of a trichome (tr), attaching to the periphery of the stomata (at). Scale bars (c-e) = 0.1 mm

A histological examination for evidence of fungal penetration as well as the presence of H_2_O_2_ was undertaken for *B. distachyon* leaf samples harvested at 7 dpi. Hyphae were noted to aggregate at leaf veins and traverse the leaf surface (Figure 3a). The production of H_2_O_2_ was associated with the site where fungal hyphae came into contact with host leaf veins as well as adjacent cells (Figure 3b, at), which corresponds with what was observed in Figure 2a where necrotic tissue developed along leaf veins. As expected *Z. tritici* hyphae exhibited brown colouration also (Figure 3b, hp). Hyphae associated with the base of trichomes stained heavily for necrosis (Figure 3a, tr), growing across the inoculated leaf surface (Figure 3a, hp). This correlates with observations in Figure 2a, tr where necrotic trichomes were frequently observed on pathogen infected tissues. Hyphae were observed growing towards and around stomatal apertures (Figure 3a, st), attaching to the stoma from the trichome (Figure 3e, tr and hp). Stomata and stomatal apertures with no evidence of fungal attachment or attempted penetration did not stain for hydrogen peroxide or fungal deposition (Figure 3c), in contrast to Figure 3a, en where stomata stained for the presence of fungal deposition and Figure 3d where the stomata stained heavily for hydrogen peroxide; likely as a result of the elicitation of a defence response following contact with the fungal pathogen.

### Differential ecotype response

**Figure 4:**
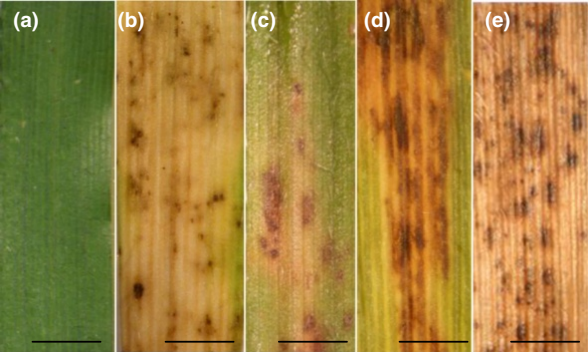
**Differential responses between the** ***B. distachyon*** **ecotypes Bd1-1, Bd12-3, Bd21-1, Bd29-1 and Bd30-1 following inoculation with** ***Z. tritici*** **at 7dpi.** Typical symptoms for each ecotype were **(a)** no disease for Bd1-1, **(b)** chlorosis with little or no development of necrotic lesions on Bd12-3, necrotic lesions surrounded by patches of yellow/red chlorosis for **(c)** Bd21-1 and **(d)** Bd29-1 and **(e)** large patches of predominately necrotic lesions under senescent tissue or green leaf material for Bd30-1. Scale bars = 0.5 cm

**Figure 5:**
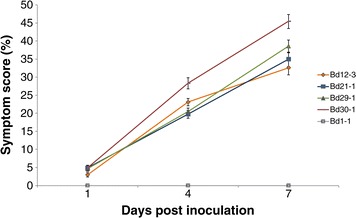
**Progression of symptoms for** ***B. distachyon*** **ecotypes Bd12-3, Bd21-1, Bd29-1, Bd30-1 and Bd1-1 following inoculation with** ***Z. tritici***. Symptom score determined as % of leaf tissue affected by symptoms (from either/all 4 symptom classes) at 1, 4 and 7 dpi. Means ± se were calculated from three biologically replicated experiments, where n = 24.

**Figure 6:**
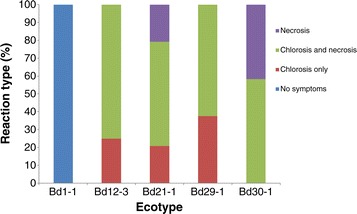
**Percentage of reaction types observed among the** ***B. distachyon*** **ecotypes Bd1-1, Bd12-3, Bd21-1, Bd29-1, and Bd30-1 following inoculation with** ***Z. tritici.*** Reaction types were classified as No symptoms, Chlorosis only, Chlorosis with necrosis or necrosis with no symptoms of chlorosis. The percentage of plants with each reaction type was calculated using three biological replicates where n = 24.

Varying degrees of symptoms and symptom progression were observed within the five *B. distachyon* ecotypes tested (Figure 4a-e). Overall symptom scores were highest for Bd30-1 (Figure 5). The rate of symptom progression was greatest up to 4 dpi, with Bd30-1 showing an 82.5% increase between 1 and 4 dpi. Between 4 and 7 dpi, symptom progression was impeded, with less than half the rate observed for Bd30-1 between 1 and 4 dpi. The fastest rate of symptom progression between 4 and 7 dpi was in Bd29-1 (46.9%) (Figure 5). All ecotypes with the exception of Bd1-1 developed similar symptoms at 1dpi (Figure 5) and reaction types followed a pattern of necrotic lesions or spots surrounded by chlorosis (Figures 4b-e and 6), however for Bd12-3, Bd21-1 and Bd29-1, 25%, 20.83% and 37.5% of the plants showed symptoms of red/yellow chlorosis only (Figure 6). For Bd30-1, all plants developed necrotic lesions, with 58.33% of the plants showing necrotic lesions (Figure 6). Bd1-1 was recorded to be completely symptomless (Figures 4a, 5 and 6) for the duration of the host-pathogen interaction. This lack of symptoms was maintained despite attempts at different methods of inoculation including physical disruption of tissues prior to inoculation (data not shown).

Sporulation was not recorded on any of the five ecotypes tested, with no pycnidia observed on tissues examined under a dissecting microscope. Similarly, the agitation of treated tissues in water did not yield any signs of *Z. tritici* growth following cultivation of the resulting eluate on PDA.

## Discussion

The present study was designed to explore the potential of using *B. distachyon* as a model host for *Z. tritici* and decipher whether this interaction might serve as a contemporary pathosystem to study mechanisms of resistance to *Z. tritici* in wheat. Phenotypically, STB symptoms in *B. distachyon* closely resembled those reported during an incompatible interaction with wheat. Initial symptoms manifested in the form of pale water soaked lesions, followed by the development of necrotic blotch-like lesions surrounded by areas of chlorosis. Significantly, sporulation could not be achieved and stomatal penetration could not be determined, indicating that *B. distachyon* is an incompatible host for *Z. tritici* (strain IPO323) infection. *Z. tritici*-related symptoms were evident on aerial parts of *B. distachyon* also, most notably the florets. In natural epidemics, STB infection of wheat heads is not frequently observed [37]. While glumes of wheat plants sometimes exhibit STB symptoms [38], attempts to isolate *Z. tritici* from seeds produced either on naturally infected plants [39] or following artificial head infections [40] have failed. After inoculation of adult wheat heads with *Z. tritici*, [41] was able to detect the fungus by microscopic observation on only 5% of the grains. These findings were corroborated by the first detection of naturally contaminated wheat seeds through PCR analysis [42], but the accurate localisation of the pathogen in the seed could not be confirmed. Thus, the presence of disease symptoms on *Z. tritici* infected florets of Bd21-1 is a curious finding.

Studies which highlight the host specificity of *Z. tritici* exist from as early as 1901 [43]. Yet, limited reports have been published since then on the interaction of *Z. tritici* with other species aside from its natural host, *Triticum aestivum*. It has been suggested by [44] that *Bromus*, *Agrostis*, *Agropyron*, *Brachypodium*, *Dactylis*, *Festuca*, *Glyceria*, *Hordeum*, *Poa*, *Secale cereale* and *Triticum* species were alternative hosts for overwintering *Z. tritici*. However, no concrete evidence has been provided to support this hypothesis. A study by [45] where 25 cereal and grass species were inoculated with *Z. tritici* showed that the fungus infected *T. aestivum, T. durum*, *T. dicoccum* and *T. compactum*, but did not infect any other species of the cereals or grasses tested. A comprehensive study by [46] described in detail the interaction between *Z. tritici* and einkorn wheat (*T. monococcum*), a diploid relative of *T. aestivum*. Out of 216 interactions tested, 98.6% were incompatible with only three compatible interactions observed. Further cytological analysis showed that resistant phenotypes blocked *Z. tritici* at three post-stomatal entry stages which correlated with the sporulation induction phenotypes. Other phenotypes severely restricted post-penetration hyphal growth of *Z. tritici*. Hence, post-stomatal entry arrest appears to be the mechanism conferring resistance to *Z. tritici* in *T. monococcum*. The observations made in this study suggest that a pre-penetration mechanism of defence could be operating against *Z. tritici* in *B. distachyon*; though the fungus was detected on epidermal tissue, Koch’s postulates for *Z. tritici* in *B. distachyon* were not confirmed. Based on our recordings, it appears that germ tubes attached to leaf structures (e.g. trichomes) and traversed epidermal cells. While attempting to gain entry to the host via stoma, their progression was arrested, which paralleled the elicitation of a defence response and the production of defence compounds such as hydrogen peroxide. The use of structures such as trichomes to anchor to and travel from is conceivable as *Z. tritici* can germinate and initiate hyphal growth on essentially almost every surface [3]. *B. distachyon* also has large trichomes, hence these may act as structures for fungal hyphae to attach to and progress across the leaf surface [47].

The results from our study of the interaction between *Z. tritici* and *B. distachyon* suggest that there is a limited level of compatibility between *B. distachyon* and *Z. tritici* and it is clear that *B. distachyon* is efficient at arresting pathogen ingress. Colonization of substomatal cavities and the intercellular space between mesophyll cells, formation of mature pycnidia in the substomatal cavities and subsequent extrusion of pycnidiospore-bearing cirrhi are the key steps in completing a successful infection cycle [4]. Though fungal sporulation could not be achieved on the ecotypes screened under even the most favourable environmental conditions it is evident that the symptoms observed on *B. distachyon* were that of *Z. tritici* and not of any other disease, due to the precautions taken in the experimental design and procedures.

Differential responses among *B. distachyon* ecotypes to biotic and abiotic stresses have previously been reported [14,25,28,30,32,48-50] indicating that naturally occurring variation may provide insights into mechanisms underlying responses to agronomically important traits. Of significance from this study was that Bd1-1 exhibited a symptomless response to *Z. tritici* strain IPO323. For Bd12-3, Bd21-1 and Bd29-1, necrotic lesions developed following chlorosis of tissues. For Bd30-1, tissues developed an early necrotic response with minimal signs of chlorosis. The interaction between Bd1-1 and *Z. tritici* strain IPO323 is likely a form of innate immunity to the pathogen, while for the other ecotypes it is possibly a form of induced defence mechanism(s) related to incompatibility between the pathogen and the host.

With the rapid uptake of RNA-Seq [51], the establishment of functional genomics tools such as virus induced gene silencing (VIGS) [52] and the advancements in genome editing techniques such as CRISPR/Cas and TALENs [53], the opportunity to study the genetic mechanisms underlying resistance to *Z. tritici* in both the host and pathogen is an exciting prospect. Encouragement can be taken from the publication of a recent study by [54] which examined the expression profile of *Z. tritici* at the early stages of the interaction with both wheat and *B. distachyon*; compatible and non-compatible hosts respectively. While expression networks common to both hosts were present, wheat and *B. distachyon*-specific transcripts were also identified, thus presenting the first study of its kind to allow for the identification of whole networks of fungal genes responsible for fungal growth and virulence in both a compatible and non-compatible host of *Z. tritici*. As segregating populations have been developed for most of the diploid inbred lines that have been researched to date [55,56]. Bulk segregant analysis of *B. distachyon* mapping populations segregating with differential phenotypes (e.g. Bd1-1 x Bd21-1) could allow for the identification of loci conferring immunity to *Z. tritici*. By combining RNA-Seq with bulked segregant analysis the identification of specific genes underlying these loci should also be possible, enabling examination of the expression profiles for candidate genes of interest while also determining gene function and location within the exome.

## Conclusions

It has been suggested by [25] that verdicts on the host or non-host status of a species cannot be based on observations made within a few ecotypes having a common genetic background, originating from a similar region. In spite of our study being limited with respect to the demographic range of ecotypes and the number of fungal isolates tested, we have identified a differential host response across the ecotypes examined. Based on the physiological assessments described here it can be concluded that the *B. distachyon* - *Z. tritici* pathosystem likely involves the activation of host defence mechanisms at the initial stages of the interaction. Incorporating the multitude of resources, tools and knowledge that are now available to the community, we encourage further investigation of *B. distachyon* as a model pathosystem with which to study this economically important cereal pathogen.
